# AEDT: A new concept for ecological dynamics in the ever-changing world

**DOI:** 10.1371/journal.pbio.2002634

**Published:** 2017-05-30

**Authors:** Peter Chesson

**Affiliations:** 1 Department of Ecology and Evolutionary Biology, The University of Arizona, Tucson, Arizona, United States of America; 2 Department of Life Sciences and Research Center for Global Change Biology, National Chung Hsing University, Taichung, Taiwan; National Centre for Scientific Research (CNRS), France, Canada

## Abstract

The important concept of equilibrium has always been controversial in ecology, but a new, more general concept, an asymptotic environmentally determined trajectory (AEDT), overcomes many concerns with equilibrium by realistically incorporating long-term climate change while retaining much of the predictive power of a stable equilibrium. A population or ecological community is predicted to approach its AEDT, which is a function of time reflecting environmental history and biology. The AEDT invokes familiar questions and predictions but in a more realistic context in which consideration of past environments and a future changing profoundly due to human influence becomes possible. Strong applications are also predicted in population genetics, evolution, earth sciences, and economics.

## Introduction

The concept of equilibrium has been the basis of prediction in ecology, as in many sciences, because the vicinity of equilibrium commonly defines the properties expected of a system. In conservation, equilibrium, as a formalization of the ancient concept of the balance of nature, has been imagined to define the essence of a system and to be treated with reverence [1,2]. In a strict equilibrium view, undeniable population fluctuations and trends [3] are treated as noise, transients, or temporary disequilibrium. Limit cycles, strange attractors, and stationary probability distributions provide alternatives to the equilibrium concept, but all suffer from the complaint that they are merely equilibria on different scales [4]. They all imply stable long-term frequencies of population states and so are incompatible with long-term climate fluctuations.

Last century, dissatisfaction with the equilibrium concept led to the introduction of models of population dynamics in which the environment is a stochastic process [5–9]. But in all cases, the environment was assumed to be stationary: any given state of the environment recurs with a predictable long-run frequency (Box 1 and S1 Text Part A). Population dynamics are then often also stationary in the long run and described by a probability distribution, the stationary distribution, which takes the place of the point equilibrium [10]. The quest for convergence on equilibrium in traditional ecology became the quest for convergence on this probability distribution, which is in essence an equilibrium probability distribution [10–13]. Human activities make us acutely aware that all populations face a future of change that is not simply a replay of some past event, which convergence on a stationary distribution necessarily implies. Also, too often, in the absence of obvious human influence, we think of what we see today at a locality as its long-term state (its “natural” state), but historical reconstruction often informs otherwise [14]. No organisms escape the influence of climate. Climate fluctuations do not have stable repetition frequencies [15,16], and the complex of factors driving climate variation gives scant expectation that they should [17]. They are nonstationary. Ecological theory should account for these major facts and not rely on the serious fiction that the present conditions are characteristic of the past. Moreover, it needs to be ready for a future of profound, anthropogenically driven change.

**Box 1. Glossary**

**Fig 1:**
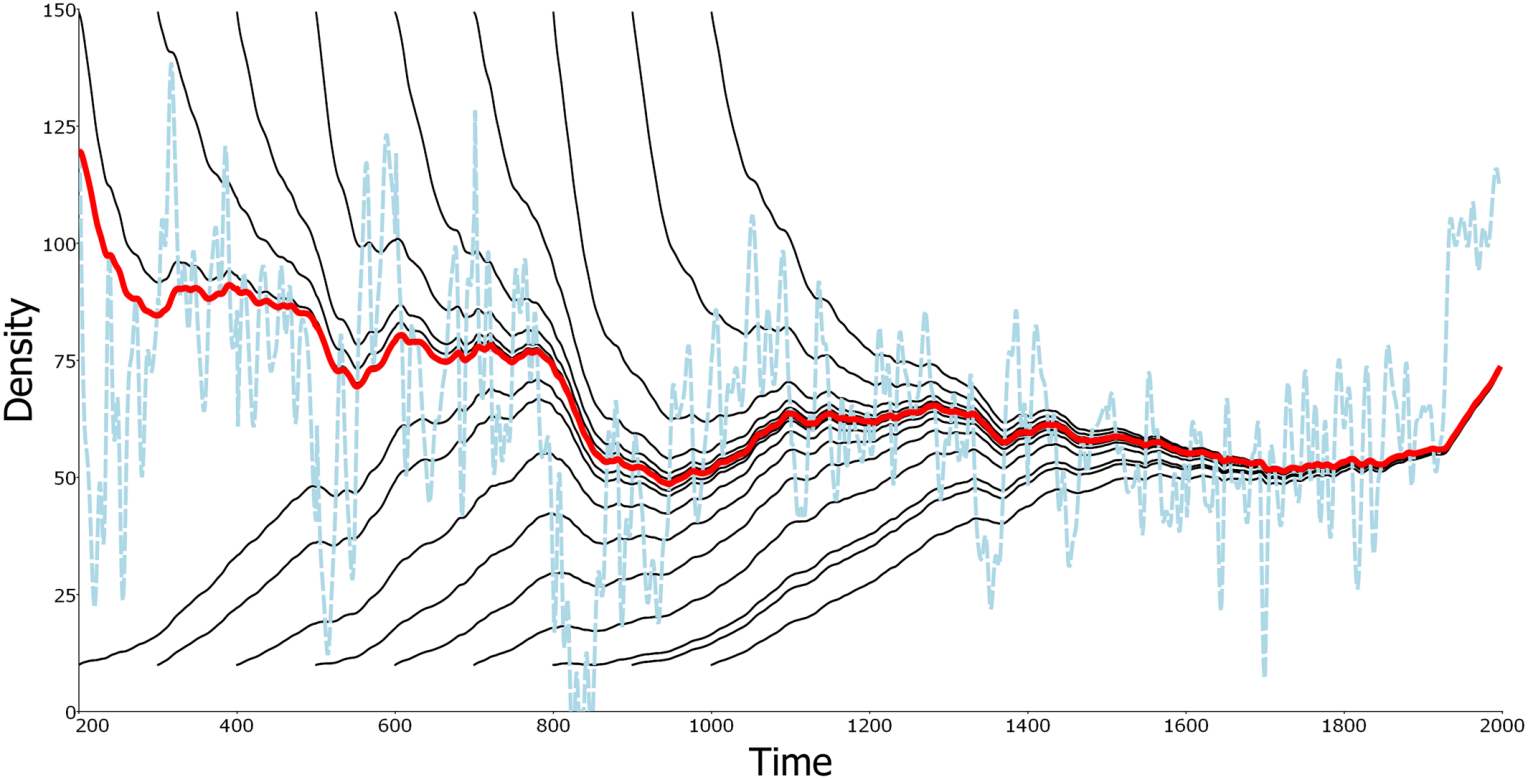
Convergence on an asymptotic environmentally determined trajectory (AEDT). Illustration using the Beverton-Holt model. Red line: the AEDT, N*(*t*); thin black solid lines: different trajectories, *N*(*t*), for different starting times, *s* (= 200, 300,…, 1000), and 2 different initial values, *N*(*s*) (upper versus lower lines). Light blue dashed line: the moving equilibrium, $N_{E(t)}^{*}$, reflecting the underlying physical environment at each point in time, which, for illustration here, depends on the reconstructed mean Northern Hemisphere temperature, 200–1995 CE (S1 Text Part C). Backward convergence is illustrated by the increasing closeness of *N*(*t*) to N*(*t*), for *t* > 1000 as the starting time, *s*, is decreased. Forward convergence is illustrated by the fact that, by 1950, most trajectories are indistinguishable from the red trajectory, N*(*t*). (For the data, see S1 Data).

AEDT: Asymptotic environmentally determined trajectory. A trajectory, N*(*t*), of the population process that is approached by other trajectories, *N*(*t*), either as the starting time, *s*, of the system recedes into the past (backward convergence) or the current time, *t*, advances into the future (forward convergence) (Fig 1).

Attractor: In nonautonomous dynamics theory, an “attractor” consists of a set of sets {*A*_*t*_}, indexed by time *t*, that are approached either in the forward or backward senses by population trajectories. If each set *A*_*t*_ consists of just 1 element, then *A*_*t*_ = {N*(*t*)}, the AEDT.

Backward convergence: In terms of the AEDT, *N*(*t*) → N*(*t*) as the starting time, *s* →–∞, for starting density, *N*(*s*), fixed at any positive finite value. In the nonautonomous dynamics literature, such convergence is known as “pull-back convergence.”

Equilibrium: The idea that *N*(*t*) remains fixed at a particular value, N*, the equilibrium point. Stable equilibrium points are of most significance, namely, values that are approached over time in the forward sense: *N*(*t*) → N* as *t* → ∞.

Forward convergence: In terms of the AEDT, *N*(*t*)–N*(*t*) → 0, as time, *t*, → ∞, for fixed starting time, *s*, and starting density, *N*(*s*).

*N*(*t*): Population density or a vector of population densities with components representing, for example, populations of different species, different ages or size classes, or populations at different spatial locations. As a function of time, *t*, *N*(*t*) defines a trajectory of the system.

Stationary environment: The idea that the environment, when viewed over a sufficiently large interval of time, will have the same statistical properties (mean, variance, autocorrelation, and frequencies of events) independently of when that interval of time starts. This is the common assumption in models with variable environments.

## Resolution

Fortunately, a foundation for rising to this challenge exists. Although not generally known, for decades, mathematicians have been developing relevant concepts and machinery in the theory of nonautonomous dynamics [18] (S1 Text Part B). Understanding of the familiar logistic model in a nonstationary environment was elucidated decades ago [19] but is not widely appreciated. Although other ecological models have been developed in this context [20,21], they have not reached ecologists; and the theory (S1 Text Part B), though powerful and elegant, has been presented mostly in mathematically abstract terms, dense with findings that are meaningful to mathematicians but less clear to others. Fortunately, an ecologically meaningful development is possible in simpler terms, which I show here.

Consider the Beverton-Holt model (Box 2), which is a discrete-time version of the logistic model. Temporal environmental variation can be represented by time-varying population parameters, which may show any pattern of change over time. The changing environment precludes convergence on a traditional equilibrium, but Box 2 shows that the population process, *N*(*t*), quite broadly converges on a time-dependent function of the environment, N*(*t*), independently of initial population densities. This convergence occurs in 2 senses. In the backwards sense, the dependence of population density, *N*(*t*), at time *t*, on the “starting” density, *N*(*s*), at time *s*, is lost as *s* recedes into the past (Fig 1). In the forwards sense, the difference between *N*(*t*) and N*(*t*) converges to zero as *t* increases (Fig 1). Like a traditional equilibrium, *N*(*t*) will equal N*(*t*) for all *t* if it is equal to N*(*t*) for any *t*. Thus, N*(*t*) is a generalization of a stable point equilibrium, but unlike a point equilibrium, it is a function of time and reflects changing environmental conditions. It is an asymptotic environmentally determined trajectory (AEDT, Box 1 and S1 Text Part B). An AEDT is a trajectory of the population process on which other trajectories converge in at least 1 of the 2 senses above. It applies even when the population process is driven by nonstationary environmental variation.

**Box 2. The nonstationary Beverton-Holt model (discrete-time logistic)**

The Beverton-Holt model of density-dependent population growth is a discrete-time model defined by the following difference equation for population density of a single-species, *N*(*t*),

$$N(t+1)=\left\{\frac{R(t)}{1+\alpha (t)N(t)}\right\}N(t).$$ (1)

Here, *R*(*t*) is the maximum finite rate of increase and *α*(*t*) is the intraspecific competition coefficient. These parameters vary over time as functions of the changing environment. The traditional equilibrium depicted in Fig 1 is $N_{E(t)}^{*}=K\left(t\right)=\left[R\left(t\right)\;–\;1\right]/\alpha \left(t\right)$ and is a function of the environmental conditions, **E**(*t*) = (*R*(*t*), *α*(*t*)), at time *t* only. It is the solution of Eq 1 for *N*(*t*+1) = *N*(*t*). The AEDT, denoted N*(*t*), is a very different quantity (Fig 1). It is most easily derived in terms of the reciprocal, 1/*N*, of *N*.

With *y* = 1/*N*, *ρ* = 1/*R*, and *a* = *α*/*R*, Eq 1 takes the linear form

y(t+1)=ρ(t)y(t)+a(t). (2)

This transformation to linearity gives an exact solution by iteration,

$$y(t)=\underset{\begin{array}{l} \text{Dependent on }\\ \text{the initial state} \end{array}}{\underset{︸}{\left[\prod_{u=s}^{t-1}\rho (u)\right]y(s)}}+\underset{\begin{array}{l} \text{Independent of }\\ \text{the initial state} \end{array}}{\underset{︸}{\sum_{u=s}^{t-1}a(u)\prod_{v=u+1}^{t-1}\rho (v)}}.$$ (3)

Because the *ρ*s are the reciprocals of the maximum finite rates of increase, they should mostly be less than 1 if the population is to persist at all. In particular, the leftmost product of the *ρ*s should converge on 0 as the starting time, *s*, recedes into the past. Under this condition, a unique asymptotic environmentally determined trajectory (AEDT) results in terms of *y*:

$$y^{*}(t)=\sum_{u=-\infty }^{t-1}a(u)\prod_{v=u+1}^{t-1}\rho (v)$$ (4)

(S1 Text Part C). Dependence on the initial state, *y*(*s*), has been lost, and only biology and the physical environment, encoded in *a* and *ρ*, remain in y*(*t*). Taking the reciprocal of y*(*t*), we have the AEDT shown in Fig 1:

$$N^{*}(t)={\left[\sum_{u=-\infty }^{t-1}a(u)\prod_{v=u+1}^{t-1}\rho (v)\right]}^{-1}.$$ (5)

**The nonstationary probability distribution**

The AEDT can also be considered as a stochastic process, and in general, it is a nonstationary stochastic process described statistically by a nonstationary probability distribution. In particular, its mean and variance change over time. Nonstationary distributions have the potential to be highly complex, but a simple form applies to the Beverton-Holt model when the parameter *ρ*(*t*) is a constant and all effects of the changing environment come from *a*(*t*) (S1 Text Part D). The AEDT of the Beverton-Holt model is approximately a Gaussian stochastic process in many cases and so is characterized by its mean, variance, and covariance functions. Regardless of the applicability of the Gaussian approximation, these moment functions can be derived in terms of the corresponding moment functions of the environment process, *a*(*t*), with mean denoted by *θ*(*t*) and variance by *φ*^2^(*t*). For simplicity, in this box, we assume no temporal environmental correlations, although arbitrary correlations present no difficulty (S1 Text Part D).

The results are simplest when expressed in terms of the reciprocal of the density, namely, y*(*t*). The theoretical mean of y*(*t*) is simply

$$\mu^{*}(t)=\frac{\hat{\theta }(t)}{1-\rho },$$ (6)

where $\hat{\theta }(t)$ is a geometric weighted average over the past mean environments, *θ*(*t* −1), *θ*(*t* −2), *θ*(*t* −3), … with weights (1 −* ρ*), (1 − *ρ*)*ρ*, (1 − *ρ*)*ρ*^2^, … reflecting Eq 4 for y*(*t*). The variance of the distribution of y*(*t*) can be written in terms of a similar weighted average,

$$\sigma^{*2}(t)=\frac{{\hat{\varphi }}^{2}(t)}{1-\rho^{2}},$$ (7)

where ${\hat{\varphi }}^{2}(t)$ is defined in terms of *ρ*^2^ by the formula ${\hat{\varphi }}^{2}(t)=\left(1\;–\rho^{2}\right)\varphi^{2}\left(t\;–1\right)\;+\;\left(1\;–\rho^{2}\right)\rho^{2}\varphi^{2}\left(t\;–\;2\right)\;+\;\left(1\;–\rho^{2}\right)\rho^{4}\varphi^{2}\left(t\;–\;3\right)\;+\ldots$. Finally, the covariance function, $\sigma^{*2}(t^{\prime },t)=\text{cov}\left(y^{*}\left(t\prime \right),y^{*}\left(t\right)\right)$, for *t* > *t*′, rounds out the first and second moments of y*(*t*):

$$\sigma^{*2}(t^{\prime },t)=\rho^{t-t^{\prime }}\sigma^{*2}(t^{\prime }).$$ (8)

These mean and variance functions have very straightforward interpretations. The environmental mean and variance, *θ*(*t*) and *φ*^2^(*t*), define the trends over time in the environmental fluctuations, i.e., they characterize its nonstationary properties. The mean and variance of y*(*t*) represent limited-time horizon averages over the past in these trends.

It is important to emphasize that the AEDT, N*(*t*), is not simply the moving value of the traditional equilibrium ($N_{E(t)}^{*}$ of Boxes 2 & 3, Fig 1), which depends only on **E**(*t*) and therefore treats the environment as frozen in time. In contrast, N*(*t*) depends on the past sequence of environmental states. Consonant with asymptotic independence from the starting density, however, the contributions of past environments diminish with their distances from the present. Nevertheless, the value of the AEDT at any given time reflects a certain amount of history. In contrast also with the moving equilibrium,$N_{E(t)}^{*}$, the observed trajectory, *N*(*t*), at time *t* can actually be expected to be near the value N*(*t*)—that is what asymptotic environmental determination implies. However, there is no expectation that *N*(*t*) should be near $N_{E(t)}^{*}$(e.g., Fig 1) unless environmental change is slow relative to population dynamics.

**Box 3. General convergence conditions**

General discrete-time population dynamics can be represented in the form

N(t+1)=F(N(t),E(t)) (9)

where *F* is some function that depends on both the population size *N* and the environmental conditions **E**. In general, *N* might actually be a vector representing multiple interacting populations or age or spatial structure in 1 or more species (S1 Text Part E), but for simplicity, here it is a single unstructured population. Key to convergence on an AEDT is that any 2 population trajectories, *N*(*t*) and *N*′(*t*), obeying Eq 9 with the same **E**(*t*) but starting at different values (*N*′(*s*) ≠ *N*(*s*)) must converge on each other. Their difference, Δ*N*(*t*) = *N*′(*t*)–*N*(*t*), satisfies the equation

$$\Delta N(t+1)=F^{\prime }(\ddot{N}(t),E(t))\Delta N(t),$$ (10)

where *F*′ is the derivative of *F* with respect to population density, *N*, and $\ddot{N}(t)$ is a number between *N*′(*t*) and *N*(*t*) given by the mean value theorem of differential calculus. Thus, Eq 10 is exact, not a linear approximation. Expressed as the difference over time, we obtain

$$\Delta N(t)=\left[\prod_{u=s}^{t-1}F^{\prime }(\ddot{N}(u),E(u))\right]\Delta N(s).$$ (11)

In standard ecological theory, stabilizing density dependence corresponds to an absolute value of *F*′ less than 1. Provided the geometric mean of these *F*′ values is ultimately bounded less than 1 either as *s* decreases (backward convergence) or *t* increases (forward convergence), the trajectories converge and become independent of their initial values. Demonstrating such convergence is potentially a more complex endeavor than traditional linear stability analyses, where *F*′ only needs to be evaluated at equilibrium, but the ecological principles are the same: convergence occurs with a preponderance of stabilizing density dependence. In other words, a perturbation of the density would be followed, after an interval of time, by density differences of smaller magnitude when there is net stabilizing density dependence. This idea generalizes to show that ecological processes that we normally expect to stabilize dynamics about equilibrium in multispecies systems and in structured populations also have roles in convergence on an AEDT (S1 Text Part E).

This demonstration provides no formula for the AEDT and allows the possibility that, although trajectories converge on each other, the starting time, *s*, continues to have a strong influence. However, given convergence on each other, whenever 1 trajectory shows backward convergence, they all do, on a unique AEDT. Moreover, convergence of Eq 11 to 0, for all pairs of trajectories as *t* increases, is, by definition, forward convergence. Although the AEDT in that case is not unique, possible definitions of the AEDT differ negligibly for large *t* (S1 Text Part B).

As emphasized, the AEDT, N*(*t*), is not the moving equilibrium, $N_{E(t)}^{*}$, defined by the equation

$$N_{E(t)}^{*}=F(N_{E(t)}^{*},E(t)),$$ (12)

but given backward convergence, the AEDT can be related to the moving equilibrium by the formula

$$N^{*}(t)=\sum_{u=-\infty }^{t-1}N_{E(u)}^{*}\left[1-F^{\prime }(\ddot{N}(u),E(u))\right]\prod_{v=u+1}^{t-1}F^{\prime }(\ddot{N}(v),E(v)),$$ (13)

where $\ddot{N}(u)$ is a number between N*(*u*) and $N_{E(u)}^{*}$(S1 Text Part E). This formula expresses the AEDT as a geometrically weighted moving average of the moving equilibrium into past with time varying geometric weights $\left[1-F^{\prime }(\ddot{N}(u),E(u))\right]\prod_{v=u+1}^{t-1}F^{\prime }(\ddot{N}(v),E(v))$ reflective of the varying strength of density dependence with time (S1 Text Part E). Note that these formulae might be applied in practice using a transformation of *N*, such its reciprocal, *y*, used for the Beverton-Holt model. Indeed, applying Eqs 11 and 13 to *y* in the Beverton-Holt model reproduces the results of Box 2.

**Fig 2:**
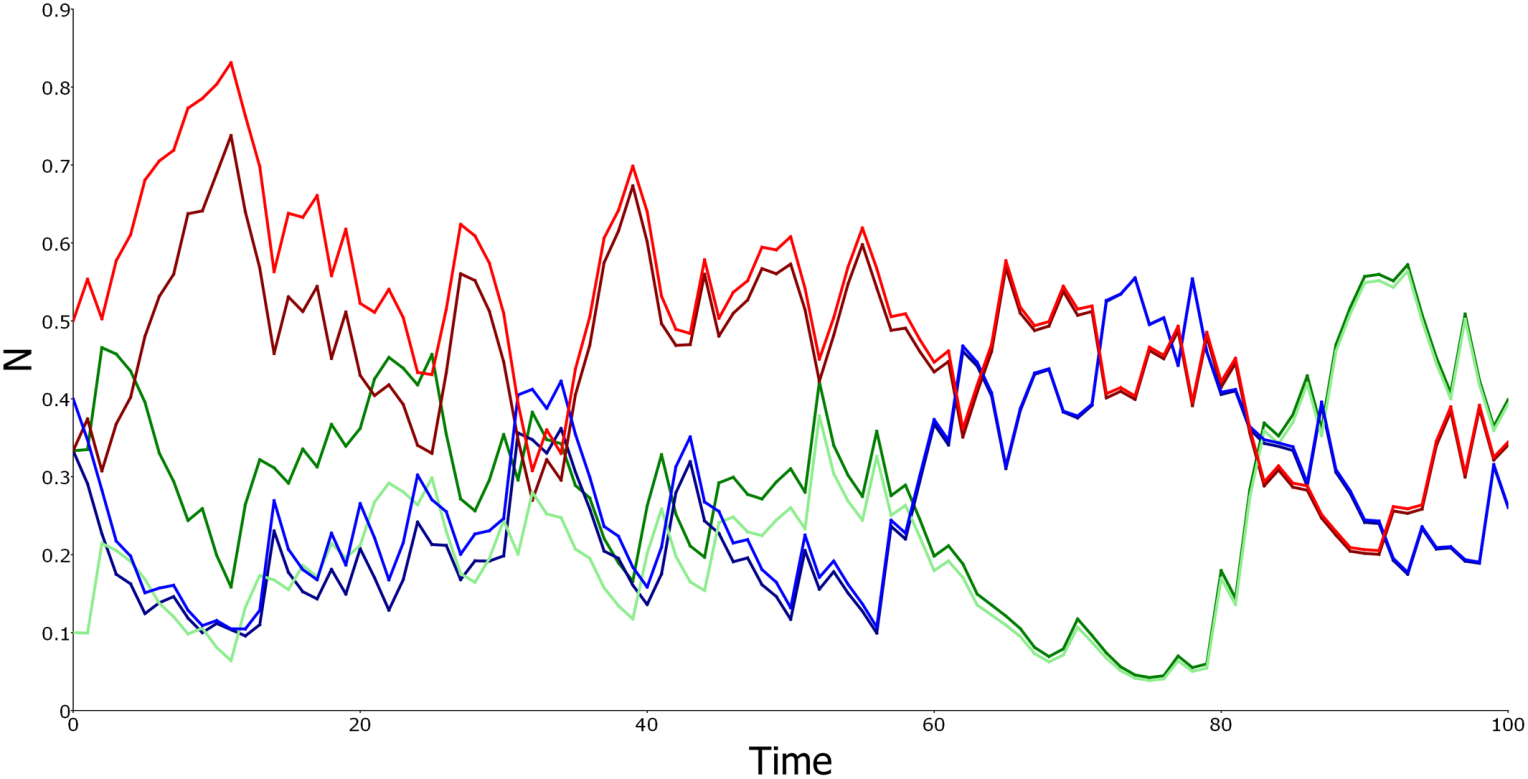
Forward convergence in the lottery model. Lines of the same color but different intensity represent the same species with different initial conditions. Although starting at very different values, the effect of the initial conditions has all but disappeared by midway through the simulation. The environmental fluctuations driving this lottery simulation are lognormal, independent between species and over time, with a linear trend creating nonstationarity. Specifically, the ln*B*_*j*_(*t*) are independent normal with means 0.05*j* + 0.002(4 –*j*)*t* and variance 1.5. For each species, *δ* = 0.25. (For the data, see S2 Data).

The AEDT concept applies not just to the Beverton-Holt model but generally in ecological models. Box 3 and S1 Text Part E show how the density-dependent feedback, critical for convergence on a traditional equilibrium, is involved with convergence on an AEDT in a variable environment. Box 4 and S1 Text Part F, on the other hand, explain how life-history phenomena behind the storage effect coexistence mechanism, which do not involve a traditional equilibrium, nevertheless give convergence on an AEDT in a variable environment, whether stationary or not, as illustrated in Fig 2. Thus, the AEDT captures an important ecological outcome, namely nonequilibrium coexistence [22], that a traditional equilibrium cannot. The AEDT shows how ideas that have formed the heart of population and community ecology can be reinterpreted in a new, more realistic context. Added to these traditional ideas, however, is the ability to analyze the effects of environmental history and their interaction with life-history processes. For example, continuing changes in United States forest composition, well-documented with pollen records, are conceptually interpretable with the AEDT concept but do not fit an equilibrium perspective [23].

**Box 4. The lottery model**

As proposed previously [6], perennial organisms competing for space in a variable environment might be modeled with the equations

$$\begin{array}{l} N_{i}(t+1)=\underset{\text{surviving adults}}{\underset{︸}{\left(1-\delta_{i}\right)N_{i}(t)}}+\underset{\begin{array}{l} \text{space given up }\\ \text{by adult death} \end{array}}{\underset{︸}{\left[\overset{n}{\underset{j=1}{\sum }}\delta_{j}N_{j}(t)\right]}}\underset{\begin{array}{l} \text{fraction of available space }\\ \text{captured by species }i \end{array}}{\underset{︸}{\frac{B_{i}(t)N_{i}(t)}{\overset{n}{\underset{j=1}{\sum }}B_{j}(t)N_{j}(t)}}},i=1,‥,n.\\ N_{i}(t):\text{ fraction of space held by species }i\\ \delta_{i}:\text{ fraction of species }i\text{ adults dying per unit time}\\ B_{i}(t):\text{ per capita juvenile production rate of species }i \end{array}$$ (14)

In this model, success in competition for space depends on the ratio *B*_*i*_(*t*)/*δ*_*i*_ for each species [6]. When environmental conditions are constant, only the species with the largest *B*/*δ* ratio persists in the long run. Unlike the models of Boxes 2 and 3, there is no stable equilibrium with all species at positive densities for any given state of the environment. All stable equilibria imply extinction of all but 1 species. Coexistence occurs, however, with the right sorts of environmental fluctuations [6], and it remains possible to demonstrate convergence on an AEDT. Fig 2 illustrates this with a simulation in the 3-species case, but in the 2-species case, with equal adult death rates (*δ*_1_ = *δ*_2_), convergence can be proved by transforming density to the log-odds scale:

$$Z_{i}(t)=\text{ln}\left\{N_{i}(t)/(1-N_{i}(t))\right\}.$$ (15)

On this scale, the difference, $\Delta Z_{i}=Z_{i}^{\prime }-Z_{i}$, between any 2 trajectories, $N_{i}^{\prime }$ and *N*_*i*_, with different starting values can be shown to decrease monotonically over time, *t*, according to the inequality

$$\Delta Z_{i}(t+1)-\Delta Z_{i}(t)\leq -\delta (1-\delta )\frac{\left[\rho (t)+\rho^{-1}(t)-2\right]\left[N_{i}^{\prime }(t)-N_{i}(t)\right]}{\delta +(1-\delta )\text{max}\left\{\rho (t),\rho^{-1}(t)\right\}}$$ (16)

where *ρ*(*t*) = *B*_*i*_(*t*) /*B*_*j*_(*t*) and *ρ*^-1^(*t*) = *B*_*j*_(*t*) /*B*_*i*_(*t*) (S1 Text Part F). The quantity *ρ*(*t*) + *ρ*^– 1^(*t*) – 2 is always positive whenever *ρ*(*t*) ≠1, i.e., whenever *B*_*i*_(*t*) ≠ *B*_*j*_(*t*). Thus, strict decreases in Δ*Z*_*i*_ require inequality between species in their responses to the environment (*B*_*i*_(*t*) ≠ *B*_*j*_(*t*)) and overlapping generations (*δ* < 1). Moreover, convergence of Δ*Z_i_* to 0 as *t* increases or *s* decreases requires *ρ*(*t*) to fluctuate about 1, thus favoring different species at different times (S1 Text Part F). These requirements are familiar issues for species coexistence by the storage effect in previous work for stationary environments [6].

The AEDT concept also resolves 1 of the discontents nearly universally seen with the traditional equilibrium idea. The system equations give convergence on a constant state, but a constant state cannot in fact be found in nature, which goes to the heart of the classic dispute between Nicholson and Andrewartha & Birch [24]. A natural system will not be at a traditional equilibrium, nor will it be close to one in most cases. The traditional equilibrium cannot be equated with the average, even in the case of a stationary environment. Although in some models the average may not be very far from the traditional equilibrium, in others, the traditional equilibrium can be grossly misleading, as models such as the lottery model (Box 4) demonstrate. In contrast, the AEDT, N*(*t*), does give predictions that can be compared directly with nature. Disagreement between N*(*t*) and observations of nature point to disagreements between nature and the model that produced the N*(*t*) prediction without being caught up in hand waving about how an equilibrium model should be interpreted in a nonequilibrium world.

Traditionally, stability of an equilibrium was interpreted as demonstrating robustness to environmental fluctuations. Even though a population or community might be continually perturbed from equilibrium, it would always be returning: equilibrium would define a central tendency for population fluctuations. However, the lottery model (Box 4), and scale transition theory more generally [25], show that this reasoning applies only to perturbing forces that are weak relative to the stability of the equilibrium, while strong perturbing forces can create new central tendencies unrelated to the traditional equilibrium [6]. These new central tendencies are captured by the AEDT to the extent that environmental fluctuations are accurately modeled (Box 4). But the AEDT also has stability properties analogous to a traditional stable equilibrium, as most clearly seen with forward convergence: a trajectory perturbed from the AEDT will return to it over time. Thus, like a traditional stable equilibrium, an AEDT with forward convergence is robust to small, infrequent perturbations outside the modeled environmental fluctuations.

Although proposed here to address nonstationary environments, the AEDT concept applies to the special case of a stationary environment too. Traditionally, analysis of a stochastic population model in a stationary environment sought a corresponding stationary probability distribution for population size, not a trajectory [12]. The statistical properties of the AEDT, however, would be described by this stationary probability distribution in that case. In the nonstationary case, there will not normally be a stationary distribution to describe the statistical properties of the AEDT, but depending on the context, a nonstationary distribution would be involved (Box 2).

Traditional equilibrium analysis focuses on determining the existence of a stable equilibrium with specific properties. In coexistence analysis, for instance, it is an equilibrium where all species have positive densities [11]. In the study of biological control, it might be an equilibrium where a pest and its natural enemy persist but with the pest below an economic threshold [26]. With convergence on a stationary distribution, the variance of the stationary distribution or its shape might be an issue [27]. The focus above has been on convergence on an AEDT, not its properties, which will, of course, be key in any examples. With general nonstationary environments, this process can be more involved than traditional equilibrium analysis. Natural questions concern the long-term prediction of N*(*t*). Will it grow indefinitely? Will it converge on 0? Or will it do something else? A nonstationary environment process, in general, can have any properties. So the issue here is determining relationships between environment process properties and AEDT properties. Formulae for the AEDT in Boxes 2 and 3 readily lead to such relationships (S1 Text Parts C, D, and E). Even though the environment is nonstationary and the AEDT is a nonstationary stochastic process (Box 2), it might nevertheless be bounded in some sense, precluding indefinite population growth or extinction. For instance, for the nonstationary lottery model, the stochastic boundedness concept applies and places probability limits on N*(*t*) (S1 Text Part G), as does the nonstationary distribution for the Beverton-Holt model (Box 2).

## Extensions and key applications

The AEDT concept is introduced here specifically to replace the point equilibrium idea but has generalizations under the general heading of “nonautonomous attractors” to also replace multiple stable points, limit cycles, and strange attractors under nonstationary environmental conditions, along with bifurcation theory for transitions between them, in the new mathematical field of nonautonomous dynamics (S1 Text Part B). Although at the present time the complexity of the field may be daunting to those not steeped in mathematics and may appear too abstract for application, numerical approaches (for example, using the ideas in Box 3) are broadly applicable and accessible (S1 Text Part E). Moreover, these fields can be expected to become more practical as examples are developed in areas of application, a few of which are given here. Nevertheless, a likely complaint is that insights from AEDT theory are too hard to come by compared with equilibrium theory. But a critical aspect of AEDT theory should be to determine when standard equilibrium analysis suffices for the problem at hand. It can do this through the information it gives on the role of environmental history and, when coupled with scale-transition theory [25], through the information it gives on the role of environmental fluctuations.

Although I have presented the AEDT concept for ecological models, it can be applied equally well in many areas of science that involve dynamics over time subject to nonstationary environments. A simple and obvious extension is to population genetics, where the dynamics of gene frequencies have many parallels to the dynamics of populations and are no less affected by nonstationary environmental change. Indeed, the lottery model (Box 4), presented here for competition between species, is also a model of competition between genotypes in an asexual population subject to temporally varying selection [28]. It and similar models have critical roles in population genetic thinking, but historical shifts between environmental states have long been a staple in the field [29]. The AEDT potentially provides a realistic way of viewing such change. Evolutionary studies likewise can make use of this concept, especially given the recognition from long-term field studies of the temporally changing selection pressures in natural populations [30]. Consequently, population morphologies are potentially described as AEDTs.

Earth sciences are often intimately involved with the environmental change yet still make use of equilibrium concepts [31]. Sometimes, the changing nature of the system is paramount, and any standard equilibrium description has little to offer, but an AEDT may apply instead. Box 5 discusses applications in hydrology. Geomorphology, a discipline in which equilibrium theories have long had a role in theories of landform development, might well find that an AEDT is a more satisfactory concept [32,33]. Finally, economic theory is replete with equilibrium ideas [34], but the environments of real-world economic systems do not follow regular patterns.

**Box 5. Applications in hydrology**

Hydrological theory provides a natural application of the AEDT beyond biology [35]. Water dynamics in nature are subject to many time-dependent effects [36], most obviously the temporal variability of rainfall. Depending on the context, hydrological models can be extremely complex, but the simplest, which are easy to illustrate, describe water exchanges between compartments. The theory of water storage provides examples with just 1 compartment (a reservoir) having highly variable inputs due to rainfall and stream flow. Following Gani [37], simple models for the amount of water stored, *S*(*t*), which arise in dam theory, can be put in the form

$$\frac{dS}{dt}=p(t)-d(t)-f(t),$$ (17)

where *p* refers to precipitation inputs, *d* defines withdrawals from the dam, and *f* defines overflow. In the existing theory, this equation would be interpreted as a stochastic differential equation, but an ordinary differential equation interpretation suffices for the purposes here. Assume here for simplicity that only *p*(*t*) is directly a function of the physical environment, with the sum of the losses *d*(*t*) and *f*(*t*) being expressible as an increasing function of *S*(*t*) alone: *l*(*S*(*t*)) = *d*(*t*) + *f*(*t*). Also, make the mild assumption that the derivative of *l* is bounded above 0 by a constant *K*. Of most importance for convergence to an AEDT is the change in the difference between the storage for 2 different starting values

$$\frac{d\left(S^{\prime }-S\right)}{dt}=-\left(l(S^{\prime })-l(S)\right)\leq -K\left(S^{\prime }-S\right),$$ (18)

with *S′ > S*. Integrating this inequality leads to the result

$$S^{\prime }(t)-S(t)\leq \left(S^{\prime }(s)-S(s)\right)e^{-K(t-s)},$$ (19)

and as *S*′(*t*) − *S*(*t*) cannot change sign (S1 Text Part H), the difference *S*′(*t*) − *S*(*t*) must approach zero as either *s* → −∞ (backward convergence) or *t* → ∞ (forward convergence). Moreover, this same result shows that *S*(*t*) is a Cauchy sequence in *s* (S1 Text Part H), and hence, there is a unique AEDT in the backward sense upon which convergence occurs both forwards and backwards, provided only that the amount of storage in the dam has an upper limit and can never be negative—features of serious models of dams. This finding is similar to the general result theorem 3.21 in [18] but does not require special assumptions about how the environment changes over time.

In ecology, population fluctuations and trends are universal, yet a standard equilibrium perspective relegates them to noise or temporary anomalies, not part of the essence of a system. With the AEDT, change is of the essence, reflecting environmental history and biology. Both forward and backward convergence imply that the reach of environmental history is limited, and the AEDT formulae in Boxes 2 and 3 show its limits and assessment within the AEDT concept. At the same time, within the relevant history, change can determine overall structure, for example, in the lottery model (Box 4), where multiple species are supported in a changing world according to the storage-effect coexistence mechanism [38], but only a single species is supported in an unchanging world. Management of ecosystems can be viewed and practiced more appropriately as managing change, including the fluctuations essential to holding it together, reflective of the role of environmental history and anticipating an environmental future strongly driven by human influence. Thus, ecology is now in a position to go beyond denying the significant reality that it needs to embrace for proper interpretation of history and a future of anthropogenically driven climate change.

## Supporting information

S1 Text Supporting information. Parts A-H.
(PDF)

S1 Data Data for Fig 1. (XLSX)

S2 Data Data for Fig 2. (XLSX)

## Abbreviations

AEDT: asymptotic environmentally determined trajectory
