# Supplementary material for: AEDT: A new concept for ecological dynamics in the ever-changing world
Source: PLoS Biol. 2017 May 30;15(5):e2002634. doi: 10.1371/journal.pbio.2002634 (PMC5466337; doi:10.1371/journal.pbio.2002634)
Supplement: S1 Text — Parts A-H. (PDF) [file pbio.2002634.s001.pdf]

Supplementary appendix S1 Text

Contents

Part A: Stationarity

Part B: The AEDT and Nonautonomous dynamics theory

Part C: The Nonstationary Beverton-Holt

Part D: The nonstationary distribution of $N^*(t)$ for the Beverton-Holt

Part E: Convergence of trajectories on each other

Part F: The lottery model

Part G: Stochastic boundedness, trajectories, and probability distributions

Part H: The dam model

Literature Cited

Part A: Stationarity

Under the strict mathematical definition, a series {**E**(t), t integer or real} is a stationary process if it has identical statistical properties under arbitrary shifts of the time parameter, t [1]. This requirement implies that events involving **E**(t) have long-run stable frequencies of occurrence. However, these frequencies might be stochastic, i.e. different for different runs of a simulation of the process even though stable over time, consistent with stationarity [1]. For an *ergodic* stationary process, asymptotic event frequencies are not stochastic and therefore are the same for every run of a simulation. Seasonal and diurnal variation might be modeled by periodic functions of time. Periodic functions are not normally called stationary processes, but they do have the property that long-run frequencies of events are stable, and they can be

formally made stationary by randomizing the starting time over one period of the process. This transformation changes no essential property, just the way the process is viewed. The working definition of stationarity used in this paper is stable long-run frequencies of events, which is a key property of stationary processes (ergodic or not), and others such as periodic processes that become stationary when viewed in the right way.

Part B: The AEDT and Nonautonomous dynamics theory

Definition of an AEDT. Given a population process, $\{N(t), t \text{ integer or real}\}$, for example, as defined in Box 2, an AEDT is a population trajectory $N^*(t)$, on which all other trajectories converge in one of the following two senses

(A) Forward convergence: $N(t) - N^*(t) \to 0$, as $t \to \infty$, whenever $N(s) > 0$ for a fixed starting time s.

(B) Backwards convergence: $N(t) \to N^*(t)$, as $s \to -\infty$, for all t, for every fixed $N(s) > 0$.

The critical issue in both cases is that the same trajectory is approached regardless of the starting value, $N(s)$, of a trajectory, provided $N(s) > 0$. For backward convergence, $N(t)$ depends not just on t but also on the starting time s, which recedes into the distant past with a fixed starting population size, $N(s)$. It is important to note that $N^*(t)$ is a trajectory of the process, i.e., follows the same dynamical rules that any trajectory $N(t)$ must satisfy, e.g. equation (9) of Box 3. For a process without immigration, $N(s) = 0$ would give permanent extinction, and so that is ruled out.

For the case of forward convergence, values of the process prior to a fixed time, s, are not considered, and the AEDT is only asymptotically unique. Indeed, any trajectory with nonzero $N(s)$ is an AEDT in that case, but forward convergence implies also that they are asymptotically the same. For backward convergence, the AEDT $N^*(t)$ is unique. Although backward and forward convergence are

distinct concepts, they both imply the same idea: after enough time has elapsed from any given initial state $N(s) > 0$, the population trajectory depends very little on that initial state, and reflects primarily the sequence of environmental states and the dynamical rules of the system. The same definitions and ideas apply to vector-valued population models representing either multiple species or structured populations subject again to the condition that initial population sizes are positive. The presence of both forward and backward convergence is most intellectually satisfying because then the unique AEDT applying for backward convergence is a natural choice for the forward direction too. However, the absence of backward convergence should not get in the way of applications when the future is the focus.

The theory of nonautonomous dynamics provides concepts and techniques that are valuable in understanding convergence on an AEDT. The theory concerns dynamical systems modeled by difference equations such as equation (9) and similar differential equations such as equation (17). The critical property distinguishing a nonautonomous system from an autonomous system is dependence of the parameter vector **E** on time. A rich theory of such systems is emerging [2-4] providing a strong foundation for study of dynamics in nonstationary environments. Although some developments treat the environment strictly deterministically, others treat it stochastically, especially in the field of random dynamical systems [5, 6], which develops valuable techniques even though the prevailing assumption in these developments is that the environment is stationary.

The simplest and best developed examples in deterministic cases are for perturbations of autonomous systems, situations transformable into autonomous systems, and cases where the environment process and population trajectories are bounded [2, 3]. In general, there is no requirement that trajectories be bounded, but it does simplify applications of the theory. However, boundedness of trajectories is not sufficient for ecological applications. For instance in the Beverton-Holt model, an important question is whether the model predicts that the population will converge on extinction or grow indefinitely. Although, no natural population can grow indefinitely, such indefinite growth nevertheless is a critical case in models in tests of sufficiency of the model to regulate the population, a question of

longstanding interest in ecology. Admitting AEDTs that allow indefinite growth is therefore important. Moreover, to understand convergence on extinction, the log scale of population size is appropriate for investigating decline of the population. On this scale, population trajectories should not be constrained to be bounded from below. Similarly, with the lottery model (Box 4 and Part F), the natural scale of analysis due to the interest in species coexistence is the log-odds scale, where trajectories are potentially unbounded both above and below. Indeed, if the environment processes are stochastic processes, there will normally be no hard bounds on the state variables, although they may be stochastically bounded, as discussed in Part G. Thus, in the Beverton-Holt model and the lottery model, the AEDTs exist but techniques that require bounded trajectories are not sufficient. Nevertheless, as we have seen here, these models are tractable without such conditions.

Nonautonomous systems theory has a strong focus on the existence and properties of attractors, which unlike the attractors of autonomous dynamical systems are functions of time [2, 3]: $\{A_t\}$ as defined in the glossary (Box 1 of the text). Zelik et al [3] review the various concepts in use. In their simplest form, when these attracting sets consist of single points, their trajectories over time define AEDTs. Otherwise, they commonly consist of multiple trajectories allowing extensions of the various autonomous attractors that contain multiple stable points, limit cycles, or are strange attractors. Attracting sets can be *forward attracting*, consistent with my use of forward convergence, but *pull back attracting* is the term in common use that corresponds to backward convergence in the usage here [2].

Part C: The Nonstationary Beverton-Holt

For the model of Box 2, the quantity

$$\hat{R}(s,t)=\left[\prod_{u=s}^{t-1}R(u)\right]^{\frac{1}{t-s}} \tag{A1}$$

is the geometric mean of the maximum finite rate of increase for the period s to $t-1$. Note that R is the

finite rate of increase at zero density. Unless the quantity (A1) is above 1 at least for large intervals of time, the population cannot persist because it means that the population would decline on average from all densities. A geometric mean bounded above 1 for large intervals of time implies also that the first product in equation (3) of Box 2, viz,

$$\prod_{u=s}^{t-1} \rho(u) \tag{A2}$$

is bounded asymptotically by a negative exponential, and thus the initial-state dependence in equation (3) of Box 2 must vanish as either s converges to $-\infty$ (backward convergence) or t converges to ∞ (forward convergence). For backward convergence, the question remains as to whether the asymptotic trajectory is finite and positive, but this outcome is simply ensured by the additional conditions that a and ρ are positive and that $a(u)$ cannot grow asymptotically as fast as exponentially in the negative direction of time. Then the quantity

$$y^*(t,s) = \sum_{u=s}^{t-1} a(u) \prod_{v=u+1}^{t-1} \rho(v) \tag{A3}$$

must converge on a finite positive value as s converges on $-\infty$, giving finite positive values for $N^*(t)$. Thus, only very mild conditions are needed to ensure convergence on a meaningful AEDT, accommodating a wide range of possibilities for nonstationary environmental conditions. The question remains as to how $N^*(t)$ might behave into the future. A natural question is whether it would predict long-term persistence of the population in some reasonable sense.

As a nonstationary environment might be anything at all, some constraints must be placed on its behavior if the question of long-term persistence is to be answered. One possible constraint is

$$a(u) \prod_{v=u+1}^{t-1} \rho(v) < Ke^{-b(t-u-1)} \tag{A4}$$

for $t > u + 1$, and some positive constants K and b. Then

$$N^*(t) > (1-e^{-b})/K \cdot \quad \text{(A5)}$$

However, uniform boundedness seems inappropriate given likely irregularities in environmental fluctuations that inevitably should lead to runs of unfavorable times. In this light, (A4) is appropriately replaced by a constraint on an average (an expected value), as follows

$$E\left[a(u)\prod_{v=u+1}^{t-1}\rho(v)\right] < Ke^{-b(t-u-1)}, \quad \text{(A6)}$$

which is then compatible with runs of unfavorable times occurring stochastically. This constraint implies that

$$E[y^*(t)] < K/(1-e^{-b}), \quad \text{(A7)}$$

which then implies that $N^*(t)$ persists in the sense of stochastic boundedness (Part G). Simply put, it means that $N^*(t)$ has a low probability of being low.

The condition (A6) is possibly too abstract for intuition on what it is says about the nonstationary environment. However, this issue disappears if it is assumed that $a(u)$ and the $\rho(v)$ have a jointly multivariate lognormal distribution; then condition (A6) can be expressed in terms of means, variances and covariances of the logs of these variables. The expected value of a product of lognormal variates is given from the multivariate normal moment generating function [7] with argument (1,1,...,1) and shows that the sum of the means of the logs, plus one half the sum of the elements of their variance-covariance matrix, needs to be less than $\ln K - b(t-u-1)$ for (A6) to apply, a condition that is relatively easy to interpret in terms of time-series properties.

The actual environmental conditions used in the simulation of Fig 1 came from a reconstruction of northern hemisphere average temperature from 200 CE to 1995 CE simply for the purpose of having the simulation driven by real data. The link between temperature and R was arbitrarily chosen to be

$$R = e^{0.1+0.01T} \quad \text{(A8)}$$

where T is the temperature anomaly. The data are the NHCRU_CPS_COMPOSITE downloaded from the National Climate Data Center (NOAA). The quantity a was arbitrarily set to the constant value 0.0001.

The continuous-time version of the Beverton-Holt model is the logistic model, which has received detailed study in at least two nonautonomous forms: deterministic [8] and stochastic [9]. Coleman [8] studied the deterministic form for case of bounded carrying capacity and intrinsic growth rate, and showed convergence on the AEDT, which he called the canonical solution. Wu and Wang [9] review more recent studies, especially the stochastic logistic, and extended these studies to the case of instantaneous jumps in population size. Although, less studied, the nonautonomous Beverton-Holt model has also appeared in previous work, but with rather different aims than the illustration here [10, 11].

Part D: The nonstationary distribution of $N^*(t)$ for the Beverton-Holt

For the Beverton and Holt model, a good approximation to the nonstationary distribution of the AEDT is available provided $\rho = 1/R$ is assumed to be a constant. Although this is in fact a serious limitation for applications of the model, it nevertheless allows a nice illustration of the properties of AEDTs as stochastic processes. The key is the representation of the reciprocal, $\{y^*(t)\}$, of the stochastic process $\{N^*(t)\}$ as a linear recursion. Then, the first and second moments of $\{y^*(t)\}$ are easily obtained in terms of ρ and the first and second moments of $\{a(t)\}$. As we shall see below, in many circumstances the distribution of the process $\{y^*(t)\}$ is approximately Gaussian, and therefore is characterized by its first and second order moments. The distribution of the process $\{N^*(t)\}$ can then be understood in terms of the reciprocal of $\{y^*(t)\}$. Moreover, $\{N^*(t)\}$ will often be approximately Gaussian also, although likely less accurately approximated in that way compared with $\{y^*(t)\}$.

The first order of business is to obtain the moments of $\{y^*(t)\}$ in terms of the moments of $\{a(t)\}$. Define

$$\theta(t) = E[a(t)], \tag{A9}$$

$$\varphi(u, v) = \mathrm{cov}(a(u), a(v)), \tag{A10}$$

$$\mu(t) = E[y(t)] \tag{A11}$$

and

$$\sigma(u, v) = \mathrm{cov}(y(u), y(v)) \tag{A12}$$

with

$$\sigma^2(t) = \sigma(t, t) \text{ and } \phi^2(t) = \phi(t, t). \tag{A13}$$

Equation (3) of the text specialized for a constant value of ρ and $y(s)$ at the fixed initial value y_{init} is

$$y(t) = \sum_{u=s}^{t-1} \rho^{t-1-u} a(u) + \rho^{t-s} y_{\text{init}}. \tag{A14}$$

On taking expected values of both sides, we obtain

$$\mu(t) = \sum_{u=s}^{t-1} \rho^{t-1-u} \theta(u) + \rho^{t-s} y_{\text{init}}. \tag{A15}$$

The covariance function now follows from the standard formula for the covariance of linear combinations of random variables (e.g. Rao 1973) and is

$$\sigma(t', t) = \sum_{u=s}^{t'-1} \sum_{v=s}^{t-1} \rho^{t'-1-u+t-1-v} \varphi(u, v). \tag{A16}$$

Under mild conditions discussed below, each of these sums converges as $s \to -\infty$ respectively to the following forms

$$\mu^*(t) = \sum_{n=0}^{\infty} \rho^n \theta(t-1-n) \tag{A17}$$

and

$$\sigma^*(t',t) = \sum_{n=0}^{\infty}\sum_{m=0}^{\infty} \rho^{n+m} \varphi(t'-1-n, t-1-m), \tag{A18}$$

where the * indicates that these moments apply to the AEDT, $y^*(t)$. The proofs that they do indeed apply, and asymptotic normality, are given below in the proofs section. Here, we further elaborate on the first and second moments, which characterize the distribution when asymptotic normality is applicable.

Expression (A17) takes on a particularly interpretable form when it is appreciated that the sequence $\{(1-\rho)\rho^n\}$ sums to one over $n = 0$ to ∞. This means that expression (A17) can be written as

$$\mu^*(t) = \frac{\hat{\theta}(t)}{1-\rho}, \tag{A19}$$

where $\hat{\theta}(t)$ is the weighted average of $\theta(t-1-n)$ using weights $(1-\rho)\rho^n$, $n = 0$ to ∞. Expression (A18) can be expressed similarly but with a double average. However, a particularly interpretable outcome is possible when $\{a(t)\}$ is independent over time but still shows nonrandom trends because the mean θ and variance φ^2 are functions of time. Expression (A19) for the mean remains unchanged, but the variance simplifies to

$$\sigma^{*2}(t) = \frac{\hat{\varphi}^2(t)}{1-\rho^2}, \tag{A20}$$

where $\hat{\varphi}^2(t)$ is the weighted average of $\phi^2(t-1-n)$ using weights $(1-\rho^2)\rho^{2n}$, $n = 0, 1, \ldots$ The covariance, for $t > t'$, is given as

$$\sigma^{*2}(t',t) = \rho^{t-t'}\sigma^{*2}(t'), \qquad \text{(A21)}$$

a form that reflects the Markovian nature of $\{y^*(t)\}$ when $\{a(t)\}$ is a sequence of statistically independent random variables.

These expressions for the mean and variance reflect the fact that $\{y^*(t)\}$ is proportional to a geometric weighted average over past values of $\{a(t)\}$. These weighted averages mean that the trends implied by the environment are smoothed over a period of the past. These trends reflect the nonstationarity of the environment, which is little constrained here. The geometric weighting reflects density dependence, i.e. interactions between individuals within the population. It gives most weight to the near past to an extent dependent on the value of ρ. The smaller ρ is, the more effect the recent past has on the moments of $\{y^*(t)\}$. On the other hand ρ near 1 means that the moments of $\{y^*(t)\}$ reflect long stretches of the past environment. In biological terms, the smaller ρ is, the stronger the density dependence, and therefore the stronger the regulation of the population to current environmental conditions.

Moments of $\{N^*(t)\}$

Exact moments of $\{N^*(t)\}$ are in general not available, but provided the coefficient of variation of $y^*(t)$ is small, good approximations are possible. To obtain these, we first write $N^*(t)$ as

$$N^*(t) = [\mu^*(t)]^{-1}\left[1 + \frac{\tilde{y}^*(t)}{\mu^*(t)}\right]^{-1}, \qquad \text{(A22)}$$

where $\tilde{y}^*(t) = y^*(t) - \mu^*(t)$. Expanding the RHS to quadratic order in $\tilde{y}^*(t)/\mu^*(t)$ leads to the approximation

$$EN^*(t) \approx [\mu^*(t)]^{-1}\left[1+\frac{\sigma^{*2}(t)}{[\mu^*(t)]^2}\right], \tag{A23}$$

with error terms of $O\left(\sigma^{*4}(t)/[\mu^*(t)]^4\right)$ under the normal approximation to $\{y^*(t)\}$ where all odd central moments are zero. The same order of accuracy is found from linear terms in (A22) leading to

$$\operatorname{cov}(N^*(t'), N^*(t)) \approx \frac{\sigma(t',t)}{\left[\mu^*(t')\mu^*(t)\right]^2}. \tag{A24}$$

The approximations correspond to the δ-method in mathematical statistics, which is developed in detail in[12]. Moreover, as shown by Rao [12], the normal approximation carries over from $\{y^*(t)\}$ to $\{N^*(t)\}$ with these moments defining the approximating process. This approximation relies on a small enough coefficient of variation of $y^*(t)$ for its reciprocal to be close to linear in $y^*(t)$.

Proofs

Convergence in mean square

To show that the moment formulate above do indeed apply to the AEDT, we need to demonstrate that the random variable $y_s(t)$, from a trajectory of y beginning at time s, with the arbitrary value y_{init} converges in mean square to $y^*(t)$. This can be done by showing that $\{y_s(t)\}$ is a Cauchy sequence in the mean square norm as a sequence in s for fixed t. From formula (A14), the mean square deviation between trajectories starting at different times can be seen to be

$$E\left[\left(y_{s'}(t)-y_s(t)\right)^2\right] = \rho^{2(t-s)}\left\{\begin{array}{l}\left[\left(\rho^{s-s'}-1\right)y_{\text{init}}+\sum_{n=0}^{s-s'-1}\rho^n\theta(s-1-n)\right]^2\\ +\sum_{n=0}^{s-s'-1}\sum_{m=0}^{s-s'-1}\rho^{n+m}\varphi(s-1-n,s-1-m)\end{array}\right\}, \tag{A25}$$

where we assume that $s > s'$, and apply the standard formulae for the means and variances of linear functions making use also of the fact that the expected value of the square of a random variable is equal to the square of its expected value plus its variance.

If the series in θ on the right hand side of (A25), and the double series in φ, both converge as $s' \to -\infty$ to values bounded respectively by $K\rho^{hs}$ and $K^2\rho^{2hs}$, for some constant K and some h between 0 and 1, for $s < 0$, then the mean square difference (A25) can be made arbitrarily small for s sufficiently large and negative. Note that this condition simply insists that $\theta(n)$ and $\phi(n, m)$ do not increase too fast as n and m approach $-\infty$. For example, they might be bounded by a constant, but boundedness of the series in (A25) by $K\rho^{hs}$ and $K^2\rho^{2hs}$ is a much weaker requirement even allowing exponential increase of $\theta(n)$ and $\phi(n, m)$ in the negative direction, with no constraints in the positive direction of time. Hence the conditions imposed here are mild. The result that (A25) can be made arbitrarily small by decreasing s, independently of s', means that $\{y_s(t)\}$ is a Cauchy sequence in the mean square norm. It follows that $\{y_s(t)\}$ converges in mean square and the limit must agree with the AEDT $y^*(t)$ with probability 1. This means also that the first and second moments of $\{y_s(t)\}$ converge, i.e. the series (A17) and (A18) converge, and these are the moments of the AEDT $y^*(t)$.

Normal approximation

The approximate normality of the process $\{y^*(t)\}$ comes from the central limit theorem. There are many forms of the central limit theorem. The standard central limit theorem for sums of independent and identically distributed random variables is not sufficient. The basic conditions for approximation by a Gaussian process are that ρ is close to 1, the variances of the $a(t)$ do not change too rapidly with time, the tails of the distributions of the $a(t)$ are constrained to not vary

too greatly in fatness, and the process $\{a(t)\}$ has long-range independence, i.e. for large values of the time separation h, $\{a(t-n), ..., a(t-3), a(t-2), a(t-1)\}$ and $\{a(t+h), a(t+h+1), a(t+h+2), ..., a(t+h+n)\}$ are approximately independent. The approximating process is then uniquely determined by the moments. If sequence $\{a(t)\}$ is not far from being a Gaussian process, then the Gaussian approximation for the process $\{y^*(t)\}$ can be expected to be highly accurate.

Rather than trying to prove this idea in the greatest generality, I simply demonstrate it for the particular case when the environment process $\{a(t)\}$ is independent over time, although otherwise nonstationary. We need a sequence of processes that converge on a Gaussian stochastic process. To do this, we have time, t, taking the rational values ..., $-2/k$, $-1/k$, 0, $1/k$, $2/k$, ..., for a positive integer k. As k goes to ∞ a continuous time process is obtained. At the same time, we replace ρ by $\rho^{1/k}$, which has the effect of restricting the amount of change that take place for successive values of t, which are $1/k$ apart, while retaining the total change possible in one unit of time. The resulting equation is

$$y_k^*(t) = \sum_{n=-\infty}^{kt-1} \rho^{t-(n+1)/k} a_k(n/k). \tag{A26}$$

The parameter ρ here is fixed, and can have any value between 0 and 1, which seems at variance with the requirement above that ρ be near 1, but note the ρ from equation (A14) has become $\rho^{1/k}$ which converges to 1 as $k \to \infty$.

We assume here that the processes $\{a_k(t)\}$ are independent over time t with

$$a_k(t) = \frac{\theta(t)}{k} + \frac{\varphi(t)}{\sqrt{k}} \varepsilon_k(t) . \tag{A27}$$

Here θ and ϕ are assumed to be continuous functions, and the processes $\{\varepsilon_k(t)\}$ are independent over time over time with mean zero and variance 1. It follows that the process $\{a_k(t)\}$ has the mean function $\theta(t)/k$, and variance $\phi^2(t)/k$. This procedure maintains constant environmental variance per unit time as k is varied, and constant mean per unit time, which are typical assumptions to obtain a central limit theorem. The temporal independence of the $\{a_k(t)\}$ means that the process $\{y^*_k(t)\}$ is a Markov process, and the to key proving that $\{y^*_k(t)\}$ is asymptotically Gaussian is to show that the transition probabilities are Gaussian. To prove this we note that

$$\tilde{y}_k^*(t) - \rho^{t-s}\tilde{y}_k^*(s) = \sum_{n=ks}^{kt-1} \rho^{t-(n+1)/k}\varphi(n/k)\varepsilon_k(n/k)/\sqrt{k}\ . \qquad \text{(A28)}$$

and that the right hand side above is statistically independent of $\tilde{y}_k^*(s)$. Therefore, the conditional distribution of $\tilde{y}_k^*(t)$ given $\tilde{y}_k^*(s)$ is simply the distribution of the RHS of (A28) shifted by $\rho^{t-s}\tilde{y}_k^*(s)$, which means that we simply need to study the asymptotic behavior of the RHS of (A28) to determine the asymptotic behavior the transition probabilities of the process $\{\tilde{y}_k^*(t)\}$.

To demonstrate asymptotic normality of the process (A28), we evaluate its characteristic function and show that it converges on the normal characteristic function. As $\varepsilon_k(t)$ has mean zero and variance 1, the theory of characteristic functions [13] (Chapter XV) implies that the characteristic function of $\varepsilon_k(t)$ takes the form

$$\exp\left(-\tfrac{1}{2}\tau^2 + \tau^2 h(\tau)\right), \qquad \text{(A29)}$$

where τ is the argument of the characteristic function and $h(\tau)$ is a function that converges to 0 as $\tau \to 0$. In the case where the $\varepsilon_k(t)$ are identically distributed, the function h is the same for every t and k, and it follows that the characteristic function of (A28) is

$$\exp\left\{\begin{array}{l} -\frac{\tau^2}{2}\sum_{n=ks}^{kt-1}\rho^{2[t-(n+1)/k]}\frac{\varphi^2(n/k)}{k}+ \\ \tau^2\sum_{n=ks}^{kt-1}\rho^{2[t-(n+1)/k]}\frac{\varphi^2(n/k)}{k}h\left(\tau\rho^{t-(n+1)/k}\varphi(n/k)/\sqrt{k}\right) \end{array}\right\}. \tag{A30}$$

The sum on the first line converges as $k \to \infty$ to

$$\int_s^t \rho^{2(t-u)}\varphi^2(u)du\,, \tag{A31}$$

while the second sum is less in absolute value than

$$\sup_{s<u\le t}\left|h\left(\tau\rho^{t-u}\varphi(u)/\sqrt{k}\right)\right|\sum_{n=ks}^{kt-1}\rho^{2[t-(n+1)/k]}\frac{\varphi^2(n/k)}{k}. \tag{A32}$$

The sum above converges to (A31) as $k \to \infty$, and the supremum converges to zero because $h(x)$ converges to zero as $x \to 0$ and φ is a continuous function and therefore bounded on finite intervals. Thus, the characteristic function (A30) of (A28) converges to

$$\exp\left\{-\frac{\tau^2}{2}\int_s^t \rho^{2(t-u)}\varphi^2(u)du\right\}, \tag{A33}$$

which is the characteristic function of a normal random variable with mean 0 and variance (A31). The variance, $\sigma^{*2}(t)$ is now given by the limiting form (A31), but the covariance formula (A21) is unmodified. The moving average interpretation (A20) is retained also, but $\hat{\varphi}(t)$ is now a negative exponential moving average rather than a geometric moving average, and $1-\rho^2$ in the denominator of (A20) is replaced by $2\beta = 2(-\ln\rho)$.

In the case where the $\varepsilon_k(t)$ are not identically distributed, the condition

$$\sup_{k,a\le t\le b} E\left[\varepsilon_k^2(t)1_{\{\varepsilon_k^2>K\}}\right]\to 0 \tag{A34}$$

as $K \to\infty$ for finite $a \le b$, guarantees that the function h above is a uniformly bounded by a function that converges to 0 as its argument converges to 0. That function replaces h in

expression (A32), and convergence on expression (A33) remains valid. The requirement (A34) is essentially the Lindeberg condition [13] for the central limit theorem.

Having demonstrated that expression (A28), viz $\tilde{y}_k^*(t)-\rho^{t-s}\tilde{y}_k^*(s)$, convergences on a mean zero Gaussian variable with variance (A31), and noting that this variable is independent of $\tilde{y}_k^*(s)$, it follows that the limiting process $\{\tilde{y}^*(t)\}$ has transition probability distribution,

$$N\left(\rho^{t-s}\tilde{y}^*(s),\int_s^t\rho^{2(t-u)}\varphi^2(u)du\right) \tag{A35}$$

i.e. the condition distribution of $\tilde{y}^*(t)$ given $\tilde{y}^*(s)$ is normal with the mean and variance above. Finally, we note that $\rho^{t-s}\tilde{y}_k^*(s)$ converges in mean square to 0 uniformly in k as $s\to-\infty$, which then serves to prove convergence of the process $\{\tilde{y}_k^*(t)\}$ on the Gaussian process $\{\tilde{y}^*(t)\}$ with transition distribution (A35). To get the process $\{y^*(t)\}$, we have to add the mean back onto $\{\tilde{y}^*(t)\}$. That mean is

$$\begin{aligned}\mu^*(t)&=\lim_{k\to\infty}\sum_{n=-\infty}^{kt-1}\rho^{t-(n+1)/k}\theta(n/k)/k\\&=\int_{-\infty}^t\rho^{t-u}\theta(u)du\end{aligned} \tag{A36}$$

with $\sigma^{*2}(t)$ given as the limit as $s\to-\infty$ of expression (A31), i.e.

$$\sigma^{*2}(t)=\int_{-\infty}^t\rho^{2(t-u)}\varphi^2(u)du\,. \tag{A37}$$

Note that the limiting Gaussian distribution allows negative values of y, which are not possible values of the reciprocal of population size. This means that the distribution only reasonably applies when the mean (A36) is large enough for the given variance for negative y values to have negligible probability. The potential for negative values are admitted by the formula (A27) which implies that negative values of $a_k(t)$ are possible for large k. Ideally, θ will be large enough

to mean that the integrals of the $a_k(t)$ over finite intervals have high probability of being positive, despite negative contributions to that integral.

Part E: Convergence of trajectories on each other

If the population model (9) of the text is replaced by a vector equation

$$\mathbf{N}(t+1) = \mathbf{F}(\mathbf{N}(t), \mathbf{E}(t)), \qquad \text{(A38)}$$

the derivative $F'(\ddot{N}(t), \mathbf{E}(t))$ is replaced by

$$\overline{\mathbf{F}'}(\mathbf{N}'(t), \mathbf{N}(t), \mathbf{E}(t)) = \int_0^1 \mathbf{F}'(h\mathbf{N}'(t) + (1-h)\mathbf{N}(t), \mathbf{E}(t))dh \qquad \text{(A39)}$$

where $\mathbf{F}'$ is the Jacobian matrix for the transformation $\mathbf{F}(., \mathbf{E}(t))$ and $\overline{\mathbf{F}}'$ is its average over the line between $\mathbf{N}'$ and $\mathbf{N}$. By the fundamental theorem of calculus, we have the vector-valued version of equation (10),

$$\Delta\mathbf{N}(t+1) = \overline{\mathbf{F}}'(\mathbf{N}'(t), \mathbf{N}(t), \mathbf{E}(t))\Delta\mathbf{N}(t). \qquad \text{(A40)}$$

It is important to note that this is an exact result, not a linear approximation. It implies

$$\Delta\mathbf{N}(t) = \left[\prod_{u=s}^{t-1} \overline{\mathbf{F}}'(\mathbf{N}'(u), \mathbf{N}(u), \mathbf{E}(u))\right]\Delta\mathbf{N}(s). \qquad \text{(A41)}$$

The issue now is whether the product of these Jacobian matrices converges on zero. In traditional local stability analyses, with an unchanging environment, the Jacobian product can be approximated adequately by the power of the Jacobian at equilibrium. The elements of these matrices all reflect feedback loops between components of the vector **N**, including both intraspecific or intraclass feedback (for structured populations) and interspecific or interclass feedback. At this level, the concerns of traditional and nonstationary analyses are the same: how do these feedbacks shrink the difference between two

population or community states overtime? For example, the traditional answer in competition studies is through stronger intraspecific than interspecific competition [14] leading to convergence on a joint equilibrium. But when the environment fluctuates, the outcome of stronger intraspecific competition than interspecific competition may emerge over time, as it well known in the lottery model (Box 4) and other models exhibiting the storage effect [14] in the stationary case. Thus, in the case of fluctuating conditions that are the focus here, the ecological mechanisms may be evident in the product of the Jacobian matrices over time, not in each individual matrix.

As remarked in Box 3, convergence of trajectories on each other does not guarantee convergence in the backwards sense on a unique AEDT. For the final piece, it is necessary to show that one trajectory converges in the backwards sense. Part H illustrates how this may be done by demonstrating that $\mathbf{N}(t)$ is a Cauchy sequence in the starting time, s. Although such a demonstration can be challenging, expression (A41) facilitates such demonstrations as is evident from the use of the corresponding expression in Part H. Given that one trajectory converges in the backward sense, they all do on a unique AEDT provided they converge on each other, as implied by convergence of (A41) to zero. In practice, such demonstrations may often be done numerically, and the simplest way of doing this is just to simulate the population process (A38) over ranges of starting times and starting values of most relevance to the application in question. Although such numerical approaches are not mathematical demonstrations, they can be sufficient for applications. Understanding of the Jacobian matrix $\mathbf{F}'$ which is critical to convergence as shown by (A41) would then provide the theoretical understanding to interpret numerical outcomes. Thus, study of AEDT's may benefit greatly from following a hybrid approach to complex models, as discussed in [15] where understanding is derived analytically, but solutions are found numerically.

Critical understanding of the AEDT is available when the moving equilibrium exists, because then the following equation applies:

$$\begin{aligned}\mathbf{N}(t+1) &= \overline{\mathbf{F}}'(\mathbf{N}(t),\mathbf{N}^*_{\mathbf{E}(t)},\mathbf{E}(t))\left(\mathbf{N}(t)-\mathbf{N}^*_{\mathbf{E}(t)}\right)+\mathbf{N}^*_{\mathbf{E}(t)} \\ &= \overline{\mathbf{F}}'(\mathbf{N}(t),\mathbf{N}^*_{\mathbf{E}(t)},\mathbf{E}(t))\mathbf{N}(t)+\left(\mathbf{I}-\overline{\mathbf{F}}'(\mathbf{N}(t),\mathbf{N}^*_{\mathbf{E}(t)},\mathbf{E}(t))\right)\mathbf{N}^*_{\mathbf{E}(t)}\end{aligned} \tag{A42}$$

where **I** is the identity matrix. This iteration leads to the formula

$$\mathbf{N}(t) = \left[\prod_{u=s}^{t-1} \bar{\mathbf{F}}'(\mathbf{N}(u), \mathbf{N}^*_{\mathbf{E}(u)}, \mathbf{E}(u))\right]\mathbf{N}(s) + \sum_{u=s}^{t-1}\prod_{v=u+1}^{t-1} \bar{\mathbf{F}}'(\mathbf{N}(v), \mathbf{N}^*_{\mathbf{E}(v)}, \mathbf{E}(v))\left(\mathbf{I} - \bar{\mathbf{F}}'(\mathbf{N}(u), \mathbf{N}^*_{\mathbf{E}(u)}, \mathbf{E}(u))\right)\mathbf{N}^*_{\mathbf{E}(u)}. \tag{A43}$$

Letting $s \to -\infty$, we see that the AEDT, if it exists in the backwards sense, must satisfy the formula

$$\mathbf{N}^*(t) = \sum_{u=-\infty}^{t-1}\prod_{v=u+1}^{t-1} \bar{\mathbf{F}}'(\mathbf{N}^*(v), \mathbf{N}^*_{\mathbf{E}(v)}, \mathbf{E}(v))\left(\mathbf{I} - \bar{\mathbf{F}}'(\mathbf{N}^*(u), \mathbf{N}^*_{\mathbf{E}(u)}, \mathbf{E}(u))\right)\mathbf{N}^*_{\mathbf{E}(u)}, \tag{A44}$$

provided the functions are sufficiently regular to allow limits to be taken inside the infinite sum. This formula then specializes to formula (13) of Box 3 in the single unstructured population case. This formula is not intended as a calculation tool — after all both the LHS and RHS involve $\mathbf{N}^*$— but to show how the AEDT relates to the moving equilibrium.

In the case of a scalar population with $\bar{\mathbf{F}}'(\mathbf{N}^*(v), \mathbf{N}^*_{\mathbf{E}(v)}, \mathbf{E}(v)) = p$, a constant, then equation (A44) rewrites as

$$N^*(t) = \sum_{u=0}^{\infty}(1-p)p^u N^*_{\mathbf{E}(t-u-1)}. \tag{A45}$$

This formula expresses $N^*(t)$ as a weighted average of the past values of $N^*_{\mathbf{E}(t)}$ with the weights coming from the geometric probability distribution, i.e. negative exponentially declining weights. A constant value of the derivative would not apply exactly as a model of a closed population, although it could apply to a sink population with immigration [16]. However, it applies to the reciprocal of N in the Beverton-Holt model in the event that R is a constant.

Part F: The lottery model

Assume that δ's are the same for two species in the lottery model of Box 4, and label these species i and j.

Consider two trajectories $N'_i(t)$ and $N_i(t)$ experiencing identical environmental conditions, i.e. having the same $B_i(t)$ and $B_j(t)$, but with $N'_i(s) > N_i(s)$. As the lottery model iteration is monotonic in $N_i(t)$, this inequality persists for all values of t. However, it is possible to show that the trajectories converge under certain conditions. To demonstrate this, we make the transformation to the Z scale (the log odds scale), where $Z = \ln(N/(1-N))$. Note that the densities in the lottery model sum to a constant, the total density of space for the two species. Without loss of generality, this can be assumed to be 1. Hence, $N_j = 1 - N_i$, in the two species case. Defining, $\Delta Z_i = Z'_i - Z_i$, the iteration (14) shows that

$$\Delta Z_i(t+1) - \Delta Z_i(t) = \\ -\int_{N_i(t)}^{N'_i(t)} \frac{\delta(1-\delta)(\rho+\rho^{-1}-2)}{[1+(1-\delta)n_i(\rho-1)][1+(1-\delta)(1-n_i)(\rho^{-1}-1)]} dn_i \tag{A46}$$

where $\rho = B_i(t)/B_j(t)$. The quantity $\rho + \rho^{-1} - 2$ is always greater 0 whenever $\rho \neq 1$, which means that the integrand in (A46) is positive whenever $\rho \neq 1$ and $\delta < 1$. Factoring $-\delta(1-\delta)(\rho+\rho^{-1}-2)$ out of the integral and noting that the denominator of the integrand is uniformly less than $\delta + (1-\delta)\max\{\rho(t), \rho^{-1}(t)\}$ gives inequality (16) of Box 4. This inequality implies that the change in ΔZ_i from one time to the next has the same sign as $N'_i(t) - N_i(t)$. It follows that ΔZ_i is monotone decreasing with time, and strictly decreasing whenever $\rho \neq 1$. For forward convergence, first of all assume that $N_i(t)$ enters a given finite interval $(v, 1-v)$, with $0 < v < 1$, infinitely often, with $\rho + \rho^{-1} - 2 > \varepsilon$ for some fixed $\varepsilon > 0$. Then inequality (16) implies that ΔZ_i must converge to zero in the forward direction, and hence ΔN_i converges to zero also. On the other hand, if there is no interval $(v, 1-v)$ entered infinitely often, either N_i and N'_i both converge on 0 or both converge on 1, in which case ΔN_i still converges to zero even though ΔZ_i need not do so. If $\rho + \rho^{-1} - 2$ were allowed to converge on zero with time, neither ΔN_i nor ΔZ_i would necessarily converge to zero. Thus, it is clear that only mild conditions are sufficient to guarantee forward convergence in the lottery model. Backward convergence follows similar principles, but the argument is more complex.

The strongest and most interesting form of convergence is of course the case where the two

species coexist. That means that also that some interval (v, $1 - v$) is entered infinitely often and therefore that ΔZ_i converges on 0. Coexistence requires the two species to be favored at different times or else one would necessarily dominate. The conditions for coexistence in the nonstationary case involve the same sorts of considerations as arise in the stationary case, namely different species being favored at different times, and also overlapping generations, viz, $\delta < 1$ (Part G). A third feature is present also, and has important role, viz covariance between environment and competition. This phenomenon occurs in the lottery model because a species strongly favored by the environment at a particular time, leading to numerous offspring, causes strong competition for the available space at that time. These are the three features that are important for coexistence by the storage effect in the stationary case [14], and we see here that they have critical roles in convergence of trajectories as well as coexistence in the nonstationary case.

Note that this analysis uses the same idea as Box 3, but uses the log-odds scale. Adding $\Delta Z(t)$ to each side of expression (A46) makes it the counterpart of expression (10) in Box 3, and proves convergence by showing that the counterpart of the derivative F' is always less than 1 and infinitely often bounded below a given constant. Thus, the counterpart of the product (11) must converge to zero.

Part G: Stochastic boundedness, trajectories, and probability distributions

Plots of population densities against time as shown in the graphs of Fig 1 and 2 are outcomes of simulations following fixed rules, driven by specific sequences of environmental states, which are themselves realizations of the environment. It might be expected that stochastic processes are involved in the generation of the environment, but once the environment has been generated, it becomes a realization of that stochastic process, and we can study the environmental sequence that has actually occurred. Thus, it can be treated deterministically, and the population dynamics that result from it, in so far as they are related to it by deterministic rules, (e.g. Boxes 2, 3 & 4 equations 1, 9 & 14) can also be treated deterministically. In the probabilistic sense, they are outcomes conditional on the environment. Properties

of the AEDT can then be determined directly in terms of the given environmental sequence. For example, the discussion above of convergence of equation (A14) depends on specific properties of the actual environmental sequence.

However, it is also possible to take a probabilistic approach where these properties are not given, but probabilistic rules generating them are given. We could then ask the probability that (A14) converges as s converges on $-\infty$ or t converges on ∞, as well as about the values it might converge on. In the stationary case, these questions have well defined answers. For example, Lemma 5.2 of [10] shows how the ergodic theorem for stationary processes guarantees both forward and backward convergence in a model like the Beverton-Holt. A nonstationary stochastic process can be anything at all, but given the simplicity of the conditions for convergence on a meaningful AEDT for the Beverton and Holt model, for a given environmental realization, is easy to see how a nonstationary stochastic process might be constrained to guarantee convergence. For example, bounding the process $R(t)$ below by a stationary process for which suitable convergence occurs, and bounding $a(t)$ above by a stationary process plus polynomial trends in both the positive and the negative direction of time identifies a broad range of nonstationary processes for which convergence on the AEDT will also occur with probability 1.

In the lottery model in the case of a stationary environment, independent from one time to the next, I show[17] how a simple linear iteration places a uniform lower bound on the probability that the population at any time is below a specific value. That bound is of the form

$$P(N_i(t) < x) < x^{\varepsilon}M, \qquad \text{(A47)}$$

for some positive numbers ε and M, which means that low population sizes have uniformly low probability of being found— a condition that is called stochastically bounded persistence. The same technique can be applied to the nonstationary case. As a simple sketch as to how this nonstationary analysis is done, assume that the environment fluctuates randomly from year to year about some nonstationary environmental trajectory, $\mathbf{E}_{\mathbf{ns}}(t)$, $t = ...-2, -1, 0, 1, 2, ...$ Conditional on $\mathbf{E}_{\mathbf{ns}}(.)$, the environment varies independently over time, but with a time-dependent probability distribution.

Following the same technique in Appendix 3 of [17], but now conditional on $\mathbf{E_{ns}}(.)$, for any given positive ε, a difference inequality can be derived:

$$E\left[\left(N_i(t+1)\right)^{-\varepsilon} \mid \mathbf{E}_{\text{ns}}(.)\right] \le E\left[\left(N_i(t)\right)^{-\varepsilon} \mid \mathbf{E}_{\text{ns}}(.)\right]\rho(t,\varepsilon)+a \tag{A48}$$

where a is a positive constant and $\rho(t, \varepsilon)$ becomes less than 1 for ε sufficiently small whenever the conditional expected growth from low density is positive, i.e.

$$\lim_{N_i(t)\to 0} E\left[\ln N_i(t+1) - \ln N_i(t) \mid \mathbf{E}_{\text{ns}}(.)\right] > 0\,. \tag{A49}$$

Like the reciprocal density in the Beverton-Holt model, inequality (A48) has the explicit solution

$$E\left[\left(N_i(t)\right)^{-\varepsilon} \mid \mathbf{E}_{\text{ns}}(.)\right] \le \left[\prod_{u=s}^{t-1}\rho(u)\right] E\left[\left(N_i(s)\right)^{-\varepsilon} \mid \mathbf{E}_{\text{ns}}(.)\right] + a\sum_{u=s}^{t-1}\prod_{v=u+1}^{t-1}\rho(v)\,. \tag{A50}$$

An average tendency for growth from low density in the lottery model is derived from fluctuations in the environment, both conditional on $\mathbf{E}_{\text{ns}}(.)$, and overall [18]. Thus, sufficient environmental fluctuations, relative to their overall tendency to favor one species over the other, can lead the products of the ρ's in inequality (A50) to converge to 0 as either s tends to $-\infty$ or t tends to ∞. This outcome requires also $\delta < 1$, or else only one of the two species could satisfy these conditions [17]. With these considerations, various scenarios for nonstationary change lead to a uniform bound

$$E\left[\left(N_i(t)\right)^{-\varepsilon}\right] < M < \infty, \tag{A51}$$

from which (A47) follows in the nonstationary case, proving stochastically bounded persistence.

Note that the condition (A6) of Part C is sufficient for condition (A51) to apply to the Beverton-Holt model with $\varepsilon = 1$ (equation A7). In addition, asymptotic bounds, both upper and lower are given by the nonstationary distribution elucidated for $y^*(t)$ of the Beverton-Holt model in Part D. These can be looked up in normal distribution tables for the asymptotic Gaussian distribution, or given limits from

Chebychev's and related inequalities directly from the moments.

Part H: The Dam Model

Box 5 leaves two loose ends. First, the sign of $S' - S$ never changes. Second, a unique AEDT is approached in the backwards sense. To see that $S' - S$ never changes sign, note that both S' and S can only change continuously, and so to change sign they would have to approach each other, become equal and cross over. As they approach each other, however, the derivative of their difference must approach zero as they are governed by the same equation, and at most they can become equal and stay equal. In most cases, however, the declining derivative of their difference means they can only approach each other asymptotically. No change between positive and negative signs is possible in any case.

To see that a unique AEDT is approached in the backward sense, assume that S' starts at the same value as S but at an earlier time s'. Indexing S by the starting time as well as the current time, we consider the sequence $\{S_s(t)\}$ as a sequence in s for fixed t with $S'(t) = S_{s'}(t)$. Equation (18) of Box 5, now implies

$$|S_s(t) - S_{s'}(t)| \le Me^{-K(t-s)}, \tag{A52}$$

where M is the maximum capacity of the dam, and therefore the maximum possible difference between S values. As a consequence, the LHS of (A52) must approach zero as $s \to -\infty$, independently of the value of s'. Thus, $\{S_s(t)\}$ is a Cauchy sequence in s and so converges on a unique value, which defines the AEDT.

Literature Cited

1. Breiman L. Probability. Menlo Park, California: Addison-Wesley; 1968.

2. Kloeden PE, Rasmussen M. Nonautonomous Dynamical Systems. Providence, RI: American Mathematical Society; 2011. 264 p.
3. Zelik SV, Chepyzhov VV. Regular attractors of autonomous and nonautonomous dynamical systems. Dokl Math. 2014;89(1):92-7. doi: Doi 10.1134/S106456241401030x. PubMed PMID: WOS:000333204700025.
4. Kloeden PE, Pötzsche C. Nonautonomous Dynamical Systems in the Life Sciences. Springer; 2013. p. 312.
5. Arnold L. Random Dynamical Systems. Berlin: Springer-Verlag; 1998.
6. Bhattacharya R, Mujumdar M. Random Dynamical Systems: theory and applications. New York: Cambridge University Press; 2007. 463 p.
7. Kotz S, Balakrishnan N, Johnson NL. Continuous Multivariate Distributions, Models and Applications: Wiley; 2004.
8. Coleman BD. Nonautonomous Logistic Equations as Models of the Adjustment of Populations to Environmental-Change. Mathematical Biosciences. 1979;45(3-4):159-73. doi: Doi 10.1016/0025-5564(79)90057-9. PubMed PMID: WOS:A1979HF84700001.
9. Wu RH, Wang K. Stochastic Logistic Systems with Jumps. Journal of Applied Mathematics. 2014. doi: 10.1155/2014/927013. PubMed PMID: WOS:000330507600001.
10. Chesson PL, Ellner S. Invasibility and stochastic boundedness in monotonic competition models. J Math Biol. 1989;27:117-38.
11. Hüls T. A model function for non-autonomous bifurcations of maps. Discrete Cont Dyn-B. 2007;7(2):351-63.
12. Rao CR. Linear Statistical Inference and its Applications. New York: John Wiley; 1973. 625 p.
13. Feller W. An Introduction to Probability Theory and its Applications: John Wiley; 1971. 669 p.
14. Chesson P. Mechanisms of maintenance of species diversity. Ann Rev Ecol Syst. 2000;31:343–66.
15. Chesson P. Scale transition theory: its aims motivations and predictions. Ecological Complexity. 2012;10:52–68.
16. Gonzalez A, Holt RD. The inflationary effects of environmental fluctuations in source-sink systems. Proceedings of the National Academy of Sciences of the United States of America. 2002;99(23):14872-7. doi: DOI 10.1073/pnas.232589299. PubMed PMID: ISI:000179224800048.
17. Chesson PL. The stabilizing effect of a random environment. J Math Biol. 1982;15:1-36.
18. Chesson P, Huntly N. Temporal hierarchies of variation and the maintenance of diversity. Plant Species Biol 1993;8:195-206.
